# Occupational Health and Safety Experiences among Self-Identified Immigrant Workers Living or Working in Somerville, MA by Ethnicity, Years in the US, and English Proficiency 

**DOI:** 10.3390/ijerph9124452

**Published:** 2012-12-06

**Authors:** Bindu Panikkar, Mark A. Woodin, Doug Brugge, Anne Marie Desmarais, Raymond Hyatt, Rose Goldman, Alex Pirie, Marcy Goldstein-Gelb, Heloisa Galvão, Monica Chianelli, Ismael Vasquez, Melissa McWhinney, Franklin Dalembert, David M. Gute

**Affiliations:** 1 Department of Civil and Environmental Engineering, Tufts University, Medford, MA 02155, USA; E-Mails: mark.woodin@tufts.edu (M.A.W.); annemarie.desmarais@tufts.edu (A.M.D.); David.Gute@tufts.edu (D.M.G.); 2 Department of Public Health and Community Medicine, Tufts University, Boston, MA 02111, USA; E-Mails: dbrugge@aol.com (D.B.); raymond.hyatt@tufts.edu (R.H.); 3 Cambridge Health Alliance, Cambridge, MA 02139, USA; E-Mail: rgoldman@challiance.org; 4 Immigrant Service Providers Group/Health, Somerville, MA 02143, USA; E-Mail: apirie@SomervilleCDC.org; 5 Massachusetts Coalition for Occupational Safety and Health, Dorchester, MA 02122, USA; E-Mail: marcy.gelb@masscosh.org; 6 697 Cambridge St. Suite 106 Brighton, MA 02135, USA; E-Mails: heloisa@verdeamarelo.org (H.G.); mchianelli@hotmail.com (M.C.); 7 Community Action Agency of Somerville, Somerville, MA 02143, USA; E-Mails: ivlostios2020@yahoo.com (I.V.); mmcwhinney@caasomerville.org (M.M.); 8 Haitian Coalition, Somerville, MA 02144, USA; E-Mail: franklindalembert@comcast.net

**Keywords:** occupational health disparities, immigrant health, community based participatory research, environmental justice

## Abstract

In this community based research initiative, we employed a survey instrument predominately developed and administered by Teen Educators to assess occupational health risks for Haitian, Salvadoran, and Brazilian immigrants (n = 405) in Somerville, MA, USA. We demonstrate that a combined analysis of ethnicity, years in the US, and English proficiency better characterized the occupational experience of immigrant workers than considering these variables individually. While years in the US (negatively) and English proficiency (positively) explained the occurrence of health risks, the country of origin identified the most vulnerable populations in the community. Brazilians, Salvadorans, and other Hispanic, all of whom who have been in the US varying length of time, with varying proficiency in English language had twice the odds of reporting injuries due to work compared to other immigrants. Although this observation was not significant it indicates that years in the US and English proficiency alone do not predict health risks among this population. We recommend the initiation of larger studies employing c community based participatory research methods to confirm these differences and to further explore work and health issues of immigrant populations. This study is one of the small number of research efforts to utilize a contemporaneous assessment of occupational health problems in three distinct immigrant populations at the community level within a specific Environmental Justice context and social milieu.

## 1. Introduction

One of the central features of the contemporary US workforce is that it is increasingly diverse. As the labor force in the US is profoundly segregated, the working conditions, occupational hazards, work benefits and level of personal protective equipment provided on the job have also been shown to vary by demographic characteristics. Such characteristics include race, ethnicity, years in the United States, English proficiency and immigrant documentation status [1,2]. Many studies show that immigrants tend to be employed in more hazardous occupations or are assigned to more dangerous tasks than native workers [3,4,5,6,7,8,9]. Immigrants have also been shown to incur higher rates of fatal occupational injuries [10], non-fatal occupational injuries [11,12], and suffer prolonged intervals of disability resulting in missing more days off work than the native born [13,14,15,16]. The dynamics of such occupational health disparities among immigrants appear to be further complicated by many factors including ineffective work training, lack of literacy, language barriers, unequal access to health care, and work place discrimination [15,16,17]. 

Given this preponderance of evidence that immigrants are disproportionately exposed to occupational hazards and risks, it is noteworthy that little effort has gone into understanding the differences that may exist among and between various immigrant groups. We pursue such an examination here through the lens of Environmental Justice to highlight the workplace inequality and injustices present among low-income immigrant workers. We recognize that this is a departure from the traditional use of the term but we hold that it accurately reflects the disparities which confront immigrant workers on a daily basis. The majority of immigrant occupational risk studies conducted in the United States have focused on Hispanic workers [7,18,19,20,21,22,23,24]. However, immigrant groups are varied and prominent differences exist between immigrant groups and even within different Hispanic sub-groups. Maxine Margolis, an anthropologist, has referred to Brazilians as the “Invisible Minority” as they may be misrepresented as being Spanish-speaking Hispanics in the US Census when they are neither Spanish- speaking nor Hispanic [25]. There are many differences between the immigrant groups which are reflected in their distinct histories, the circumstances under which they migrated, resettled to a new place, found work, and earned a livelihood [26,27]. Both years residing in the US and English proficiency are good indicators of assimilation and increased well-being. A few studies have documented health disparities within the immigrant population by considering years in the US [28,29,30,31] and English Proficiency [32,33,34]. 

The ascertainment of distinct immigrant group characteristics at the community level are largely absent in existing occupational health surveillance systems [3,35,36]. The national report authored by the United States Bureau of Labor Statistics demonstrated that a full third of the respondents did not disclose their race or ethnic origin [37]. This incomplete characterization of the socio-cultural background of the affected populations highlights one of the central difficulties in understanding the occupational health burden found among immigrant workers. The lack of comprehensive information on the immigrant occupational health experience is acutely felt by both community and regulatory agencies that either provide services to immigrant populations or promulgate occupational health standards [3]. Community based participatory research (CBPR) is increasingly being employed to gather data at the community level to better understand the occupational health experiences of immigrant populations [38,39]. 

Our study location, Somerville, Massachusetts has witnessed a steady growth in its immigrant population over the past three decades. The Boston Metropolitan Area, which includes Somerville is termed as a “continuous” immigrant gateway community [40]. According to the American Community Survey, over a quarter of Somerville’s residents (25.2%) are immigrants [41]. This is substantially greater than the corresponding US immigrant proportion of 12.5%. One-third of this foreign born population is made up of recent immigrants who entered Somerville in 2000 or later. The most prominent immigrant groups in Somerville originate in El Salvador, Haiti, Brazil, Mexico, Guatemala, Honduras, China, India, Korea, Vietnam, and Nepal [41]. This study highlights the occupational health experience of three predominant immigrant groups in Somerville, Massachusetts–Brazilian, El Salvadoran and Haitian by their years in the US and English proficiency. 

## 2. Methods

### 2.1. Study Design and Data Collection

One of our project goals was to establish a community capability for gathering and disseminating information on work and health among the immigrant populations in Somerville. The project relied upon a core group of community based partners, Immigrant Services Providers Group/Health (ISPG/H), Community Action Agency of Somerville (CAAS), Haitian Coalition (HC), Brazilian Women’s Group (BWG) and benefitted from their ability to identify and interact with immigrant populations. The Haitian Coalition predominantly provided access to the Haitian population, CAAS to the Hispanic population and the Brazilian Women’s Group to the Brazilian population [42]. Since there were no Asian community organizations of sufficient scale, our work did not specifically target this population, however we did not exclude them from the surveys. The project also benefited from partnerships with the Cambridge Health Alliance (CHA), the predominant health care provider in Somerville, and the Massachusetts Coalition for Occupational Safety and Health (MassCOSH) for technical assistance in occupational safety and health. The Community Partners have contributed significantly in designing the study, gathering data, collaborating in the selection of variables for analysis, and also in enriching the discussion concerning the study results. The immigrant occupational health surveys were administered to gather descriptive data to better understand the socio-demographic characteristic of immigrants in Somerville, their occupational backgrounds and to identify needs relative to issues involving health and occupational health and safety. 

A survey was designed and administered by a group of bilingual (English and the target languages of our populations of interest) Teen Educators who were recruited and supervised by the Haitian Coalition and CAAS in partnership with the Massachusetts Department of Public Health (MADPH), MassCOSH, and the Tufts University researchers. Teen Educators are community youth workers who were employed by some of our community partners to help address various public health and environmental health issues in the community. This work provided the youth workers with valuable leadership experience and addressed pertinent issues of concern to the community such as tobacco use control and Brownfield development. In our present study we adapted these existing youth programs to complement our research goals and also to offer educational outreach services to the community that was previously unavailable to the community on various occupational health and safety issues. A total of 22 teens participated in the development and administration of the surveys which began in 2006 and ended in 2009. 

The survey was comprised of 23 structured questions divided into four segments—basic demographics, occupation, access to health resources, and health risks. The demographic variables included country of birth, years in the US, English proficiency, gender, and age. The occupational variables included type of work, and occupational classification. All the occupational variables were coded by employing the US Bureau of Labor Statistics Standard Occupational Classification (SOC) system. Access to health variables included work training, health and safety training, knowledge of the existence of Workers’ Compensation, as well as having health insurance and a doctor. The health risk variables included self-reported hazards at work, and self-reported injuries suffered at work, and self-reported health problems due to work. An important structural feature of the surveys is that one of the health variables, the presence of a regular doctor, was asked only among a smaller proportion of the respondents (n = 277) out of 405 total respondents. This question was added as a supplement to the original questionnaire to identify and satisfy the immigrant needs at an occupational health fair. 

The surveys were performed in an informal conversational style with time of completion averaging 30 min. The surveys were administered face-to-face by Teen Educators and researchers in Spanish, Portuguese, Haitian Creole or English. The potential study subjects were briefed on the survey’s purpose and if the respondents were 18 years of age or older and responded positively to the question, “Do you live or work in Somerville, Massachusetts?” an oral consent was sought from them for study participation. The bilingual Teen Educators then translated the survey questions into the appropriate target language in real time and were encouraged to discuss the questions with the respondent rather than simply reading them verbatim.

Convenience sampling was implemented by selecting participants and performing interviews at events that were sponsored by the partnering community based organizations such as at yearly influenza clinics organized by the ISPG/H and two occupational health fairs conducted in conjunction with the CHA. No names were ascertained nor was documentation status asked. This methodological decision was taken upon the strong and unanimous advice of the community based organizations involved in the work. As a means of increasing capacity for research within the community based organizations associated with our project the project coordinators were trained as Independent Investigators within the framework of the Tufts Institutional Review Board protocols. The benefits and challenges posed through such enhanced capacity within the community based organizations have been described previously [43]. The survey questionnaires and all study procedures were reviewed and approved by the Tufts University Social, Behavioral and Education Research Institutional Review Board (IRB). 

### 2.2. Data Analysis

Statistical analysis was performed using SPSS 17.0. The analytical plan included performing cross tabulations and chi square statistics to identify significant associations. Differences by ethnicity, years in the US, and English proficiency for any of the responses were considered statistically significant if the two-tailed p-value was less than 0.05. 

In our analysis, Country of Birth correlated with Years in the US and Years in the US correlated with English Proficiency. The overall coefficient of 0.2 was not high enough to cause any significant collinearity issues. We therefore conducted logistic regression controlling for Country of Birth, Years in the US, and English Proficiency to identify potential confounding in our results within this study.

Our study sample (n = 405) constitutes a little over two percent of the total immigrant population in Somerville as estimated by the 2000 US Census. The survey participants, consistent with the US Census data, show great diversity with respondents from 34 different countries including the United States. The respondents who answered affirmatively to a question of being US born (n = 59) were removed from the analysis. Although US born persons with immigrant parents sometimes think of themselves as immigrants we decided to restrict our analysis to only immigrant (non-US born) respondents (n = 346). In this analysis, the country of birth categories was Haiti, El Salvador, Brazil, Other Hispanics and Others. The category “other Hispanics” includes respondents born in Puerto Rico, Mexico, Belize, Honduras, Guatemala, Colombia, Peru, Venezuela, Ecuador and Dominican Republic. The category “others” includes immigrants from Asia, Africa and Portugal. Recent immigrants were defined as immigrants who have been in the United States for less than 15 years. Established immigrants were defined as immigrants who have been in the US for over 15 years at the time of their participation in the survey. 

Three major immigrant groups are represented in the survey based on their country of birth; Brazil (n = 98, 28%), Haiti (n = 83, 24%), and El Salvador (n = 74, 21%). The other Hispanics category (n = 40, 12%) and the others category (n = 51, 15%) completed the categorical data.

## 3. Results

### 3.1. Bivariate Analysis by Country of Birth

Table 1 shows that country of birth was significantly correlated with the number of years in US (χ^2^ = 67.8, *p* = 0.045). Salvadoran respondents were in the US longest, with 34% in the US for over 15 years. Brazilians respondents, in contrast, were in the US for the shortest time with 32% in the US for less than three years and only 8% in the US for over 15 years. 

**Table 1:** Cross-sectional survey results obtained from self-identified immigrant workers, Somerville, Massachusetts—2006–2009.

	Haitian	El Salvador	Brazil	Other Hispanic	Other	χ^2^
						*p Value*
**Years in US**						
1–3 years	11 (14%)	5 (7%)	31 (32%)	9 (23%)	10 (20%)	67.838 (0.045)
4–9 years	29 (36%)	26 (35%)	52 (54%)	10 (25%)	18 (36%)	
10–15 years	29 (36%)	18 (24%)	5 (5%)	8 (20%)	17 (34%)	
>15 years	12 (15%)	25 (34%)	8 (8%)	13 (10%)	5 (10%)	
**English Proficiency**						
Yes	51 (61%)	36 (49%)	48 (49%)	31 (78%)	34 (67%)	14.127 (0.153)
No	32 (39%)	38 (51%)	50 (51%)	9 (22%)	17 (33%)	
**Occupational Classification**						
Management & Professional	5 (6%)	2 (3%)	8 (8%)	2 (5%)	3 (6%)	131.902 (0.700)
Sales & Office	13 (17%)	2 (3%)	5 (5%)	4 (10%)	10 (20%)	
Service	32 (41%)	32 (44%)	35 (37%)	19 (48%)	15 (30%)	
Production & Transportation	10 (13%)	21 (29%)	1 (1%)	4 (10%)	4 (8%)	
Construction & Maintenance	2 (3%)	4 (6%)	44 (46%)	4 (10%)	3 (6%)	
Unemployed	16 (20%)	11 (15%)	3 (3%)	7 (17%)	15 (30%)	
**Work Training**						
Yes	50 (76%)	39 (64%)	37 (39%)	22 (65%)	34 (85%)	34.815 (0.981)
No	16 (24%)	22 (36%)	57 (61%)	12 (35%)	6 (15%)	
**Health and Safety Training**						
Yes	41 (58%)	35 (51%)	29 (32%)	16 (42%)	29 (69%)	19.938 (0.981)
No	30 (42%)	33 (48%)	61 (68%)	22 (58%)	13 (31%)	
**Knowledge of Massachusetts Workers’ Compensation Law**						
Yes	39 (53%)	29 (41%)	30 (33%)	17 (46%)	28 (68%)	16.932 (0.324)
No	35 (47%)	42 (59%)	62 (67%)	20 (54%)	13 (32%)	
**Health Insurance**						
Yes	55 (72%)	38 (56%)	32 (34%)	20 (59%)	29 (74%)	48.701 (0.786)
No	11 (15%)	23 (34%)	56 (59%)	13 (38%)	5 (13%)	
Don’t know	10 (13%)	7 (10%)	7 (7%)	1 (3%)	5 (13%)	
**Access to Doctor**						
Yes	44 (98%)	38 (70%)	25 (68%)	23 (72%)	33 (81%)	15.279 (0.113)
No	1 (2%)	16 (30%)	12 (32%)	9 (28%)	8 (19%)	
**Injured at Work**						
Yes	16 (24%)	22 (36%)	28 (30%)	12 (32%)	6 (15%)	6.798 (0.420)
No	52 (77%)	39 (64%)	66 (70%)	25 (68%)	35 (85%)	

Some differences were observed between the immigrant groups and access to occupational health services (work training, health and safety training, knowledge of Workers’ Compensation Law, health insurance, and access to a doctor) and health risks (hazards at work, health problems due to work and injuries at work) though these observations were not statistically significant under a chi-square test. Other Immigrants and Haitians in general reported better access to occupational health services and lower health risks than Brazilians, Salvadorans and other Hispanics. Brazilians overall had the lowest access to work training, health and safety training and knowledge of Workers’ Compensation. Though this may have contributed to higher health risks, Brazilians did not report the highest (30%) injuries at work compared to Salvadorans (36%). 

### 3.2. Bivariate Analysis by Years in the United States

Table 2 shows the chi-square analysis of the number of years an immigrant has been in the US and outcome variables. English proficiency and gender significantly varied by time in the US. English proficiency increased with the number of years in the US. Half of the recent immigrants did not speak English while 81% of the immigrants who had been in the US over 15 years spoke English. Most of the recent immigrants were male. 

Table 2 also shows a significant relationship between the number of years that immigrant respondents have been in the US and the occupational sector (management & professional, sales and office, service, construction & maintenance, production & transportation and unemployed) they work in. Recent immigrants were more likely to be employed in construction or to report being unemployed. Recent immigrants under three years in the US were less likely to be in professional jobs and production jobs. 

Significant associations were observed between the number of years an immigrant has been in the US and health and safety training, knowledge of Workers’ Compensation, having health insurance and access to doctor. We noted a crucial difference between immigrants who have been in the US for less than three years and those who have been in the US longer in our data (Table 2). Immigrants who have been in the US for less than three years seemed especially vulnerable and were, in particular, less likely to report health and safety training, knowledge of Workers’ Compensation, health insurance, and access to doctors as compared to immigrants who have been in the US over three years. This vulnerability among the most recent immigrants however did not result in self-reported health risks. On the contrary, a negative association between years in the US and injuries at work (χ^2^ = 7.1, *p* = 0.035) was observed in this study. No difference in injuries was noted among immigrants who have been in the US for less than 15 years.

We further conducted a chi-square for linear trend analysis to examine the relationship between years in the US and the outcome variables. Table 2 shows a significant linear relationship between years in the years in the US and English proficiency, health and safety training, having health insurance, knowledge of Massachusetts Workers’ Compensation Law, and injuries at work. All the variables above except access to a doctor showed significant linear trends across years in the US. The general pattern was increasing frequency of each variable as years in the US increased. Possessing health insurance and having knowledge of Massachusetts Workers’ Compensation Law both showed especially strong linear trends across years in the US.

**Table 2:** Years in the US and demographic, occupation and health outcomes obtained from self-identified immigrant workers, Somerville, Massachusetts 2006–2009.

	1–3 years	4–9 years	10–15 years	>15 years	χ^2^*p Value*	χ^2^ linear trend *p Value*
**English proficiency**						
Yes	33 (50%)	65 (48%)	48 (62%)	51 (81%)	21.3 (0.001)	0.041
No	33 (50%)	70 (52%)	29 (38%)	12 (19%)		
**Gender**						
Male	41 (62%)	62 (46%)	28 (41%)	24 (39%)	8.4 (0.010)	*NA*
Female	25 (38%)	72 (54%)	40 (59%)	37 (61%)		
**Occupational Classification**						
Managerial/Professional	1 (1%)	7 (5%)	5 (7%)	7 (11%)	58.3 (0.040)	*NA*
Sales & Office	6 (9%)	16 (12%)	7 (9%)	5 (8%)		
Service	19 (29%)	53 (40%)	36 (49%)	24 (39%)		
Production/Transport	1 (1%)	12 (9%)	14 (19%)	13 (21%)		
Construction/Maintenance	24 (36%)	26 (20%)	4 (5%)	1 (2%)		
Unemployed	15 (23%)	17 (13%)	8 (11%)	11 (18%)		
**Health and safety training**						
Yes	15 (27%)	65 (54%)	36 (52%)	32 (54%)	13.0 (0.014)	0.017
No	41 (73%)	56 (46%)	33 (48%)	27 (46%)		
**Knowledge of Massachusetts Workers’ Compensation Law**						
Yes	16 (27%)	57 (47%)	36 (52%)	32 (53%)	11.4 (0.004)	0.005
No	44 (73%)	65 (53%)	33 (48%)	28 (47%)		
**Health insurance**						
Yes	21 (33%)	62 (51%)	50 (78%)	39 (66%)	31.8 (0.001)	0.001
No	35 (56%)	44 (36%)	12 (19%)	15 (25%)		
Don’t know	7 (11%)	16 (13%)	2 (3%)	5 (8%)		
**Access to Doctor**						
Yes	20 (62%)	65 (78%)	47 (82%)	28 (82%)	5.4 (0.056)	0.069
No	12 (38%)	18 (22%)	10 (18%)	6 (18%)		
**Injured at Work**						
Yes	13 (23%)	30 (25%)	16 (24%)	24 (42%)	7.1 (0.035)	0.041
No	43 (77%)	88 (75%)	50 (76%)	33 (58%)		

### 3.3. Bivariate Analysis by English Proficiency

Table 3 shows the results of the analysis of English Proficiency. English proficiency was significantly associated with the occupational sector. The majority of the construction workers had no English proficiency. More immigrants in professional jobs and sales work spoke English. Significant associations were observed between English proficiency among immigrant groups and work training, health and safety training, knowledge of Workers’ Compensation, health insurance and access to a doctor. Immigrants who were less proficient in English reported that they received less occupational health services compared to the immigrants who are proficient in English. 

**Table 3:** English proficiency and occupation and health outcomes obtained from self-identified immigrant workers, Somerville, Massachusetts 2006–2009.

	Yes	No	χ^2^*p Value*
**Occupational Classification**			
Management & Professional	16 (8%)	4 (3%)	15.9 (0.001)
Sales & Office	24 (12%)	10 (7%)	
Service	76 (40%)	57 (40%)	
Production & Transportation	21 (11%)	19 (13%)	
Construction & Maintenance	22 (12%)	35 (24%)	
Unemployed	33 (17%)	19 (13%)	
**Work Training**			
Yes	118 (69%)	64 (52%)	8.3 (0.004)
No	54 (31%)	59 (48%)	
**Health and Safety Training**			
Yes	103 (56%)	47 (37%)	10.8 (0.001)
No	80 (44%)	79 (63%)	
**Massachusetts Workers’ Compensation Law**			
Yes	105 (57%)	38 (29%)	22.4 (0.001)
No	81 (43%)	91 (71%)	
**Health Insurance**			
Yes	117 (65%)	57 (43%)	16.8 (0.001)
No	46 (26%)	62 (47%)	
Don’t know	15 (9%)	14 (11%)	
**Access to Doctor**			
Yes	101 (84%)	62 (70%)	6.3 (0.012)
No	19 (16%)	27 (30%)	
**Hazards at Work**			
Yes	71 (40%)	59 (49%)	4.1 (0.050)
No	76 (43%)	50 (42%)	
Don’t Know	29 (17%)	11 (9%)	
**Injured at Work**			
Yes	40 (23%)	44 (35%)	5.3 (0.021)
No	135 (77%)	82 (65%)	

Immigrants who were not proficient in English reported more hazards at work and injuries at work. 

### 3.4. Binary Logistic Regression

Table 4 shows the results of binary logistic regression analyses of the dichotomous outcome variables Work Training, Health and Safety Training, Knowledge of Workers’ Compensation, Access to a Doctor and Injured at Work. Our referent group for the country of origin was always the other immigrant category since descriptive analyses showed that this group tended to have more favorable outcomes in all categories. The referent category for years in the US was immigrants in the US over 15 years since the descriptive analysis showed significant difference in health risks between immigrants who have been in the US and those who have been in the US less than fifteen years. We examined several combinations of predictor variables and found that Ethnicity (entered as a categorical variable), years in the US, and English Proficiency were all important predictors of each outcome variable. 

**Table 4:** Odds ratio and *P* values by ethnicity, years in the US and English proficiency.

	Ethnicity					Years in the US	English Proficiency
	Other	Haiti	Salvador	Brazil	Other Hispanic		
Work Training	Ref	0.57 (0.295)	0.36 (0.054)	0.13 (0.001)	0.32 (0.05)	0.86 (0.667)	1.78 (0.033)
Health and Safety Training	Ref	0.64 (0.295)	0.56 (0.175)	0.25 (0.001)	0.31 (0.014)	1.07 (0.833)	2.01 (0.007)
Knowledge of Workers’ Compensation	Ref	0.55 (0.160)	0.36 (0.021)	0.27 (0.002)	0.33 (0.025)	1.21 (0.556)	2.78 (0.001)
No Doctor	Ref	0.09 (0.027)	1.53 (0.413)	1.73 (0.309)	1.83 (0.296)	0.61 (0.341)	0.50 (0.062)
Injuries at Work	Ref	1.65 (0.352)	2.26 (0.127)	2.20 (0.119)	2.39 (0.135)	2.65 (0.005)	0.47 (0.008)

Logistic regression (Table 4) showed that Brazilians, Salvadorans and Other Hispanics reported lower work training, health and safety training, and Knowledge of Workers’ Compensation compared to Other immigrants (comprised of Asians, Africans and Europeans). Brazilians, Salvadorans, and other Hispanics were between 87% and 63% less likely to have received work training. No significant difference in work training was observed between those in the US over 15 years and those in the US for less than 15 years. Immigrant workers proficient in English were 1.7 times more likely to have received work training than those not proficient in English. Likewise, Brazilians were 75% and other Hispanics were 69% less likely to have reported having received health and safety training compared to the reference group. Immigrants proficient in English were twice as likely to have reported receiving health and safety training. The odds of reporting the knowledge of Workers’ Compensation was significantly less among Brazilians, Salvadorans, and other Hispanics compared to the referent group. They were 73% to 64% less likely to have reported knowledge of Workers’ Compensation. English proficiency was found to be a strong predictor of knowledge Workers’ Compensation as well. The odds of reporting knowledge of Workers’ Compensation were over three times higher in immigrants who are proficient in English. Haitians were negatively correlated and were 91% more likely to have a doctor compared to other immigrants. 

Compared to the referent group, no other ethnic group had statistically significantly higher odds of having a work-related injury, however, the odds ratio for El Salvadorans, Brazilians, and Other Hispanics were all about equally elevated with odds ratios between 2.2 and 2.4. Years in the US and English Proficiency were both significantly predictive of presence of work-related injury. Compared to immigrants who had been in the US for 15 years or more, those in the US less than 15 years were 2.65 times as likely to have had a work-related injury (*p* = 0.005). English proficiency was significantly protective against work-related injury with immigrants having good proficiency, more than 50% were less likely to have incurred a work-related injury compared to those immigrants without good English proficiency (OR = 0.47, *p* = 0.008). Additional regression analyses of the outcomes Hazards at Work and Health Problems due to Work showed no significant associations with any combination of predictor variables (data not shown). 

## 4. Discussion

Few studies have concurrently looked into the occupational and health experiences among different immigrant populations, (in our case- Brazilian, Haitian and Salvadoran groups) at the community level. In this study, we found that demographic factors relevant to the immigrant experience (Country of origin, years in the US, and English proficiency) may explain the occupational health disparities (injuries, health and safety training, knowledge of Workers’ Compensation, health insurance and access to a doctor) among immigrants in Somerville. Based on our sample, ethnic differences alone did not explain all the reported occupational health outcomes noted between the immigrant groups. Such differences were more effectively explained by taking the years an immigrant has been in the US and English proficiency into account when considering occupational health disparities among immigrants. We also found a significant difference in the type of occupation an immigrant holds shaped by length of tenure of these immigrant groups in the US and by English proficiency. More recent immigrants who have been in the US for less than three years tended to be in highly unstable occupations such as construction, while only 2% of the established immigrants were in construction. Construction workers were the least likely to be proficient in English as well. An analysis exploring the complexity of the distribution of low-wage immigrant occupations will form the basis of a separate paper (Author, Under Review). 

The differences between Brazilians and Salvadorans are especially interesting. Brazilians were the most recent immigrants in the study while Salvadorans have been in the US longest—for over 15 years. The associations however were not always linear as one would assume, long term residency among Salvadorans did not necessarily improve their English proficiency and occupational health experiences—access to occupational health services and health risks at work compared to other immigrants. In some cases long term residency of over 15 years showed increased reports of health risks as reported among Salvadorans. The very recent immigrants who have been in the US for less than three years were also vulnerable and had lower access to all occupational health services than immigrants residing in the US for over three years. English proficiency had a positive influence on access to occupational health services and a negative influence on health risks. English proficiency did increase with years in the US especially after ten years however one cannot assume based on this information alone that all immigrant groups progressed equally in terms of proficiency in English and will also report better access to occupational health experiences. Only 49% of both Salvadorans and Brazilians spoke English and Salvadorans were twice as likely to report health risks as Brazilians. 

Some of the results in this study are consistent with other studies among immigrant populations. The demographic characteristics of the immigrants in this study are similar to other reports such as the Immigrant Learning Center report on “Massachusetts Immigrants by Numbers” and the City of Boston Statistics on immigrants in Boston [44,45]. Uriarte and colleagues showed that Salvadorans had the lowest educational attainment (a variable that we did not explore) among the Hispanic population in Boston [27] which may explain the lower English proficiency in this immigrant group and low access to occupational services. There are very few occupational health studies done among Salvadorans which makes it difficult to calibrate the validity of this finding [21]. Haitians in Somerville have also been shown to be better educated than African Americans [46] which may explain this group’s better access to occupational health services though they are more recent immigrants than Salvadorans. Alternatively, this improvement may result from greater access to advocacy and service programs offered to the Haitian community in Somerville. 

In comparing our study with previous research among the Brazilian population in Massachusetts, we find that Brazilians were primarily employed in the service and construction sectors, and had the least amount of work and health and safety training [25,47,48,49,50]. In our study, 68% of the Brazilian workers received no health and safety training compared to 80% in the Collaboration for Better Work Environment for Brazilians report, while Marcelli reported 75% had no health insurance as compared to 60% in our survey [49]. Consistent with our study, Setia *et al.* and Asanin and Wilson show that recent immigrants were less likely to have a doctor and knowledge of Workers’ Compensation laws [28,31]. English proficiency positively influenced health outcomes in this study as well as in Smith *et al.*, Premji *et al.* and Bonauto *et al.* that reported employment in jobs with higher physical demands, and disabilities among people not proficient in English [32,33,34]. 

Glazier *et al.* using census and hospitalization data in Toronto (Ontario, Canada), showed that hospital use and serious morbidity were highest in areas with high rates of recent immigration [30]. De Castro *et al.* found more adverse health outcomes among recent Filipino immigrants with regard to the impact of work on their well-being [29]. One would assume that the number of years in the US would positively influence or lower the reports of work related injuries. However this observation is at odds with our result in that, established immigrants self-reported a higher injury history. The reasons for an increased injury experience among more established immigrants are less clear. This could be explained as a “healthy immigrant effect” where a consistent decline in health status has been observed among immigrants the longer they remain in the US [51,52]. Antecol and Bedard show that immigrants converge to American standards within ten to fifteen years [53]. The increase in injuries among the more established immigrants could also be because of the cumulative burden of exposures that the established immigrants have suffered after a longer interval of US work experience or the increased competency, and aptitude to report these injury experiences. Alternatively, the increased injury burden reflects the lack of upward mobility among these established immigrants (Author, Forthcoming). In our survey we did not ask the participants if the reported work related injury happened within the past year, which remains a major obstacle in interpreting our data and might help explain this increase in the reports of injuries among more established immigrants. However, it could also be that the increase in injuries among the immigrants who have been in the US over 15 years is being driven by the established Salvadoran population who are the least proficient in English compared to other immigrant groups. 

## 5. Limitations

Limitations include modest sample size, and an unknown but likely variable reluctance on the part of some respondents to disclose information regarding health and occupation. A study done by Franzini and Fernandez-Exquer found that Spanish speaking immigrants had lower levels of trust and higher levels of perceived victimization than the English speaking native born [54]. Another potential issue is the quality of the data gathered. Although the Teen Educators were trained to administer the questions consistently, variability in communicating the questions among interviewers, and reticence among study participants to discuss occupational and health issues with a minor could introduce errors. Another potential limitation is selection bias. The age distribution of the survey population might reflect the overt or subtle choices made by the Teen Educators in terms of their own comfort level with regards to interacting with potential respondents. The age distribution of the participants in the survey shows that 16% of the participants were between the ages of 18–20 years which suggest a possible over-selection of people closer to their age. 

The survey respondents were obtained from convenience samples. About half the sample was collected from population who attended flu clinics and health fairs and the other half was reached through the efforts of the participating community groups and through focus groups conducted within the respective communities. One concern here is that our respondents may constitute a more “mainstream” subsection of the overall ethnic population known to our community based partners leaving out the most vulnerable segment of the immigrants living and working in Somerville. Hence the reported prevalence is not necessarily generalizable and may in fact be conservative. 

Our study is not exempt from the drawbacks of serial convenience sampling among a given population that runs the risk of enrolling study participants more than once. No study participants reported that they had previously participated in the survey to our knowledge. As an added quality control measure we also checked for duplicate responses to selected survey responses such as country of origin, age, and gender. This analysis revealed no exact duplicate responses among the immigrant population. 

The health outcomes in this study are self-reported and thus the results may contain some undetermined degree of inaccuracy. Worker expectations processed through culture specific beliefs about personal health may also contribute to a biased recall of health-related events. However, we think the information reported here and the processes set in motion have value despite these limitations. 

## 6. Strengths and Contributions

This is one of the few studies that attempts to provide a concurrent assessment of multiple immigrant populations in a single city. This allows us to view the occupational health experience of specific immigrant population such as Brazilian, Salvadoran and Haitian within the same local milieu. We have published more detailed findings by occupation on this same study population previously [55]. We also believe that the examination of these, similar but distinct, immigrant profiles through the lens of Environmental Justice yielded a clearer picture of some of the factors which are associated with work-related health risks among US immigrants. Immigrants in informal job sectors and job arrangements are hard to reach. They may not be willing to participate in research studies due to concerns over documentation status, and possible employer reprisal [7]. We were able to overcome some of these obstacles due to a collaborative strategy which fully engaged our community partners in the development and implementation of the survey. Our data collection strategies were designed to minimize respondent burden. We benefited from the deep community knowledge possessed by our partners in fashioning outreach initiatives. Overall, the employment of the Teen Educators was successful in breaking down barriers that historically have made immigrant workers a hard-to-reach population. 

The Teen Educators, in turn, were also able to disseminate occupational health awareness in the community as part of their activities and, in addition, made contributions to their broader community as a result of engaging in advocacy activities and community outreach. We also launched the Vida Verde Co-Op, a cooperative of Brazilian housecleaners using green cleaning products, which received substantial media publicity and further increased our visibility in the community and raised occupational health and safety awareness among the immigrant community [42]. The combination of these accomplishments and capacities greatly benefited our project through the creation of a high level of trust, interaction, and communication. 

## 7. Conclusions

This community based survey provides a contemporaneous view of the occupational health disparities present among the principal immigrant populations living and working in Somerville, MA, USA by ethnicity, years in the US and English proficiency. We found that a combined analysis of ethnicity, years in the US and English proficiency better characterized the occupational experiences of immigrants in our community. Our study also showed the nuances in analyzing these variables individually. While English proficiency improved with the years in the US in our sample it did not mean that Salvadoran population who have been in the US the longest were the most proficient in English or that they had lower health risks. While years in the US and English proficiency adequately explained the occurrence of health risks, the ethnic background of the population was a useful variable to better contextualize and understand the results. Brazilians, Salvadorans, and other Hispanic, all of whom have been in the US varying length of time, with varying proficiency in English language had twice the odds of reporting health risks due to work. 

We recommend the initiation of larger studies which employ community based participatory research to confirm these differences and to further explore these ideas. Addition of an education category and the more specific inquiry into the occupational health experience and health risks suffered in the past year will further strengthen the results. We also feel that these studies need to be repeated every five years to document the occupational health disparities among this population and to measure the progress in services provided to the immigrant population.

We believe that to effectively reduce occupational health risks in immigrant populations, interventions will have to be designed that are targeted to address immigrant group-specific risk factors at the community scale. Few studies have documented and compared the concurrent occupational health experience among different immigrant groups in the same local milieu. Understanding these socio-cultural and occupational dynamics is of critical importance in the development of more effective risk reduction programs for immigrant workers. 
